# A Sex-Dependent Cannabinoid CB1 Receptor Role in Circadian Tearing of the Mouse

**DOI:** 10.1167/iovs.65.14.10

**Published:** 2024-12-04

**Authors:** Natalia Murataeva, Kyle Yust, Sam Mattox, Wenwen Du, Alex Straiker

**Affiliations:** 1 The Gill Institute for Neuroscience, Indiana University, Bloomington, Indiana, United States; 2 Department of Psychological and Brain Sciences, Indiana University, Bloomington, Indiana, United States; 3 Program in Neuroscience, Indiana University, Bloomington, Indiana, United States

**Keywords:** cannabinoid, CB1, circadian rhythm, tearing, lacrimation

## Abstract

**Purpose:**

We have shown that cannabinoid CB1 receptors regulate both salivation and tearing, but for tearing, this regulation is sex dependent with opposing effects by sex. We investigated a potential interplay of circadian and cannabinoid regulation of tearing.

**Methods:**

We measured cannabinoid and circadian regulation of tearing in CD1 strain mice as well as CB1 receptor protein expression using immunohistochemistry.

**Results:**

We now report that CD1 strain mice have a circadian variation in basal tearing, differing by sex in terms of phase and amplitude. The amplitude of circadian variation in females is substantially dampened relative to males. Male CB1 receptor knockout mice do not differ from strain controls, but in female CB1 knockouts, the amplitude is enhanced and resembles that of WT males. This increased tearing is mimicked by the CB1 antagonist SR141716 (4 mg/kg, intraperitoneally [IP]), suggesting that tonic CB1 activation dampens female circadian tearing. Consistent with this, the cannabinoid receptor agonist CP55940 (0.5 mg/kg, IP) decreases tearing during the rest phase but increases tearing during the active phase in females. CB1 protein expression also differs by sex. While both males and females have CB1 receptors in parasympathetic inputs to the lacrimal gland, in female lacrimal glands, CB1 is also detected in myoepithethial cells.

**Conclusions:**

Mice have a sex-dependent circadian cycle of tearing. The endogenous cannabinoid signaling system appears to mediate some circadian effects, albeit in a sex-dependent manner and via distinct cellular targets.

Tearing occurs via dedicated tubuloacinar lacrimal glands that produce the aqueous layer of the tear film and are essential to ocular health and vision.^1^^–^^4^ Abnormal tearing can strongly affect quality of life, and the annual burden of dry eye disease on US health care is substantial.^5^ Multiple factors can enhance the risk of developing the disorder, especially age and gender, as well as some common medications and autoimmune diseases.^2^ Tearing is under complex regulation by neuronal and other factors.^6^

Most animal species are affected by the daily light/dark cycle and adapt physiologically and behaviorally to this cycle.^7^ Many circadian effects are controlled by the suprachiasmatic nucleus (SCN) and by the expression of clock genes in peripheral tissues.^8^ Few studies have examined circadian variation of tearing parameters, although one study reports altered cytokine levels in secreted tears in human subjects.^9^ Investigations of tear flow in humans can be challenging partly because human eyes are closed during the sleep phase.^9^^,^^10^ Consequently, animal models have proven valuable, with a study in horses indicating that tearing varies significantly by time of day.^11^ Mice have also proven a valuable model in animal research, including the study of circadian regulation,^12^ albeit with some important caveats discussed below.

The cannabinoid signaling system consists of receptors (CB1 and CB2), endogenous lipid messengers, and enzymatic machinery that synthesizes and metabolizes these messengers “on demand.”^13^ This cannabinoid signaling system plays multiple roles in the eye, regulating retinal signaling,^14^ ocular pressure,^15^ and migration of corneal epithelial cells.^16^ We have additionally reported that CB1 receptors regulate both tearing and salivation^17^^,^^18^ but with a notable difference: in both males and females, CB1 receptor activation reduces salivation but has sex-dependent effects on tearing. CB1 receptor activation reduces tearing in males but increases tearing in females. The present study is an outgrowth of efforts to understand the basis for these sex differences. In the present study, we examined circadian regulation of tearing and its interplay with the cannabinoid signaling system.

## Methods

### Animals

Experiments were conducted at Indiana University. All mice used for experiments were handled according to the guidelines of the university animal care committee and in accordance with the ARVO animal statement. Adult mice (age 3–6 months) were kept on a 12-hour (08:00–20:00) “reverse” light/dark cycle and fed ad libitum. C57BL/6 (C57) mice were purchased from Jackson Laboratories. CB1 KO on a CD1 background and CD1 strain-mates were kindly provided by the laboratory of Dr. Ken Mackie (Indiana University, Bloomington, IN, USA). The experiments described here independently investigated male and female mice. Conventional CB1 null mice (CB1^–^^/^^–^) were originally received from Dr. Catherine Ledent (Catholic University, Leuven, Belgium).^19^

### Measurement of Tearing

As described previously,^17^ to measure tearing in mice, a phenol red thread was positioned at the rear corner of each eye (the lateral canthus of the conjunctival fornix) of an awake behaving mouse for 10 seconds. Tears discolor the threads for later quantification—more tearing results in a longer discolored portion of the thread. The length of the discolored portion can therefore be taken as a measure of tearing, allowing comparison of experimental conditions to baseline conditions or changes over time. The values from both eyes were averaged for a given animal. In contrast to our previous study, mice were repeatedly handled before these experiments to accustom them to measures of tearing. As a consequence, it was possible for a single experimenter to both hold the mouse and obtain a tear sample. For experiments that tested the effect of drug treatments (CP55940, SR141716, and tetrahydrocannabinol [THC]), a baseline tearing value was obtained, after which animals were injected with drugs intraperitoneally (IP, 0.5 mg/kg [CP55940], 4 mg/kg [THC, SR141716]). One hour after injection, a second experimental tear measurement was taken.

Tearing measurements were taken in a room with regulated humidity (35%), temperature (22°C), and light (100 lux).

### Statistics

**Figure 1.**
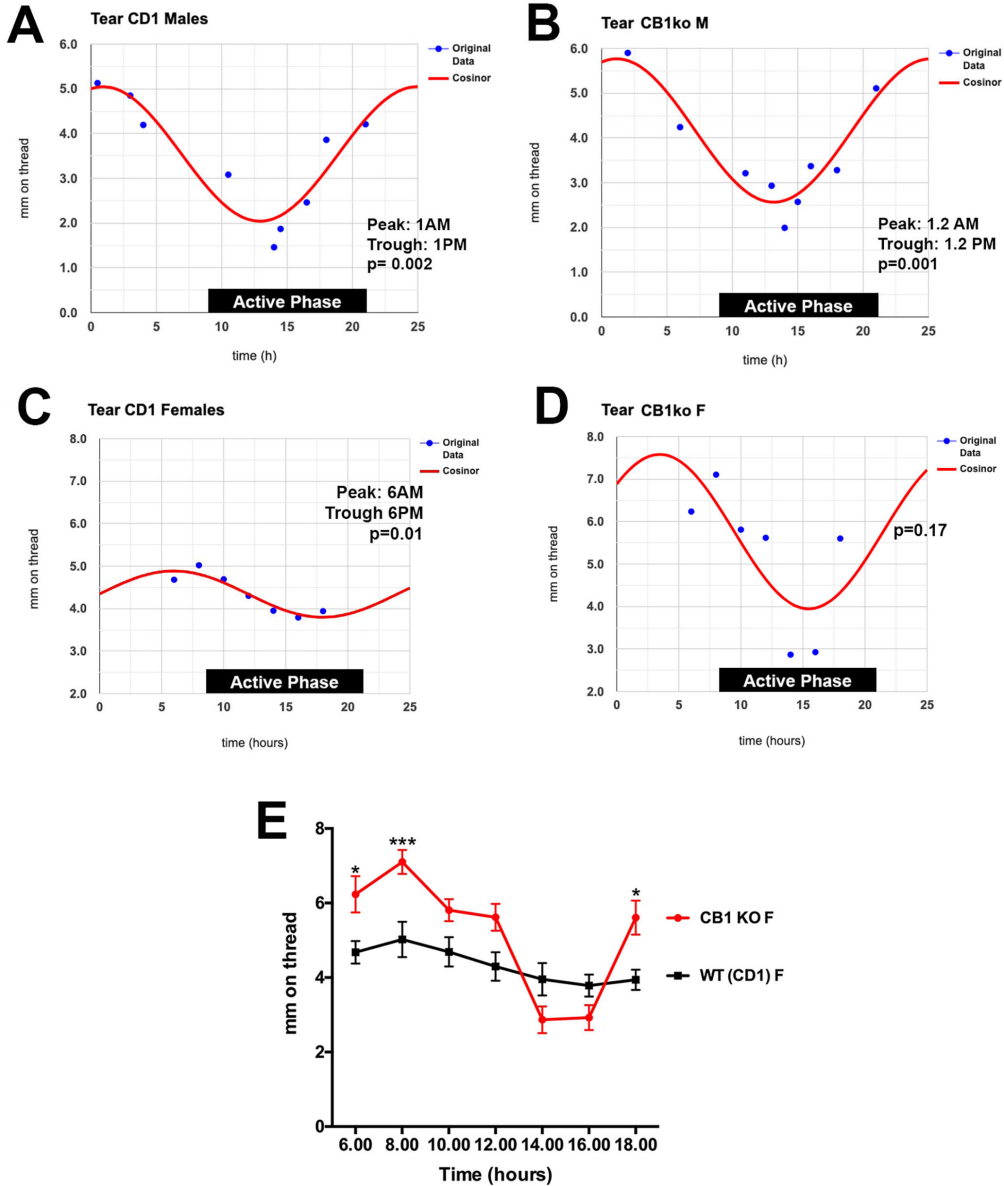
Sex-dependent circadian effects of CB1 deletion on tearing. (**A**) Cosinor analysis shows a circadian variation of tearing in CD1 male mice, *n* = 15. (**B**) Male CB1 KO mice on a CD1 background have the same profile, *n* = 12. (**C**) Female CD1 strain mice have a delayed phase relative to males, *n* = 21. (**D**) Cosinor does not fit the responses in female CB1 KO mice (*P* = 0.17, *n* = 17). (**E**) Analysis of female CB1 KO versus strain controls shows an increase in tearing during the rest phase. *P < 0.05 at 6 AM and 6 PM, ***P < 0.005 at 8 AM, two-way ANOVA with Šídák post hoc test.

For drug treatments, experimental values were compared to baseline using a paired *t*-test. Cosinor statistics (e.g., zero-amplitude test) were calculated at the cosinor.online site. For the comparison of multiple time points (Fig. 1E), we employed a two-way ANOVA with the Šídák post hoc test. Sample sizes are listed in the respective figure legends.

### Immunohistochemistry

For immunohistochemistry, lacrimal glands were fixed in 4% paraformaldehyde for 45 minutes at 4°C, then placed sequentially in 10% and 30% sucrose in PBS overnight before being suspended in OCT (Thermo Fisher Scientific, Waltham, MA, USA) in a 15-mL clear plastic tube to allow for precise three-dimensional orientation of the sample. The tube was then submerged in cold (–80°C) ethanol to rapidly freeze the sample. Fixed/frozen glands were sectioned on a Leica cryostat (Leica Microsystems, Wetzlar, Germany). Sections were then mounted on Superfrost Plus slides (Thermo Fisher Scientific). Slides were blocked with BSA, followed by treatment with primary antibodies (in PBS, Triton-X, 0.3%) for 1 to 2 days at 4°C. Where secondary antibodies were required, a second staining with secondary antibody (∼4 hours at room temperature [RT]) was done after washing off the primary antibody. Primary antibodies were CB1 (1:300, host guinea pig, cat. Af530; Frontier/Nittobo), choline acetyltransferase (ChAT) (1:300, host goat, cat. AB144P, lot 2211015; Millipore), and phalloidin-594 (cat. A12381; Life Technologies). Phalloidin was employed as a counterstain that identifies intra-acinar ducts. Secondary antibodies were labeled with the Alexa 488 or Alexa 647 (Thermo Fisher Scientific). Slides were then mounted with mounting media containing 4′,6-diamidine-2′-phenylindole dihydrochloride to visualize nuclei (Fluoromount; Sigma-Aldrich, St. Louis, MO, USA). Images were acquired with a Leica TCS SP8 (Leica Microsystems)**.** Images were processed using FIJI (available at https://imagej.net/Fiji/downloads) and/or Photoshop (Adobe, San Jose, CA, USA) software. Images were modified only in terms of brightness and contrast.

### Drugs

THC and SR141716 were obtained through the NIDA Drug Supply Program. CP55940 was obtained from Cayman Chemical (Ann Arbor, MI, USA).

## Results

### Male and Female CD1 Strain Mice See a Pronounced Circadian Variation in Basal Tearing, With a Difference in Phase and Amplitude

In testing the circadian cycling of tearing, the first question was which strain of mice to employ. Our previous study had largely been limited to C57BL/6 mice,^17^ but this strain has deficiencies in melatonin and so lacks many phenotypic effects of clock regulation.^12^ We tested C57BL/6 strain tearing during the middle of the active and rest phases. As expected, this strain saw only minimal changes in tearing (females: rest phase [noon] [mm ± SEM]: 4.6 ± 0.62; active phase [midnight]: 3.9 ± 0.56, *n* = 11; males: rest phase: 3.9 ± 0.39; active phase: 3.5 ± 0.44, *n* = 12).

As an alternative, we chose CD1 strain mice, not least because our CB1 knockout mice are on this background. Testing them, we found that these mice had a clear circadian cycling in their tearing. Male wild-type (WT) CD1 mice had a clear circadian cycle peaking at around 1 AM, with a trough at 1 PM (Fig. 1A, *df* = 2, 6, *F* value = 20.7, *P* = 0.002 by zero-amplitude test). Tearing varied ∼2.5-fold, with a low of 2 mm and a high of 5 mm. Female CD1 mice had a similar day/night cycle to their tearing, but their phase differed by 5 hours, peaking at 6 AM (Fig. 1B, female WT: *df* = 2, 4, *F* value = 17.7, *P* = 0.01 by zero-amplitude test). Importantly here, the amplitude of variation in females was dampened relative to males, with a low of 3.7 mm and a high of 4.9 mm.

### Female but not Male CB1 Knockouts See an Increased Amplitude of Circadian Tearing

We next tested CB1 knockout (KO) mice, also on a CD1 background strain. The male CB1 KO cycle did not differ appreciably from WT, peaking shortly after 1 AM (1.18 decimal hours; *df* = 2, 6; *F* value = 26.9, *P* = 0.001) and with a trough at just after 1 PM. The situation for female CB1 KOs was very different. As shown in Figure 1D, the cosinor analysis for female CB1 KOs did not fit the responses (*P* = 0.17, Fig. 1D). A direct comparison of the data points shows that tearing was more pronounced in the CB1 knockouts during time points that appear to correspond to acrophase (i.e., the peaks appeared to be higher, Figure 1E; *P < 0.05 at 6 AM and 6 PM, ***P < 0.005 at 8 AM, two-way ANOVA with Šídák post hoc test). The phase appeared to be unaltered, with peaks at 8 AM and a trough in the later afternoon.

### CB1 Receptor Activation Produces Opposite Effects on Tearing in Females, Depending on the Time of Day

**Figure 2.**
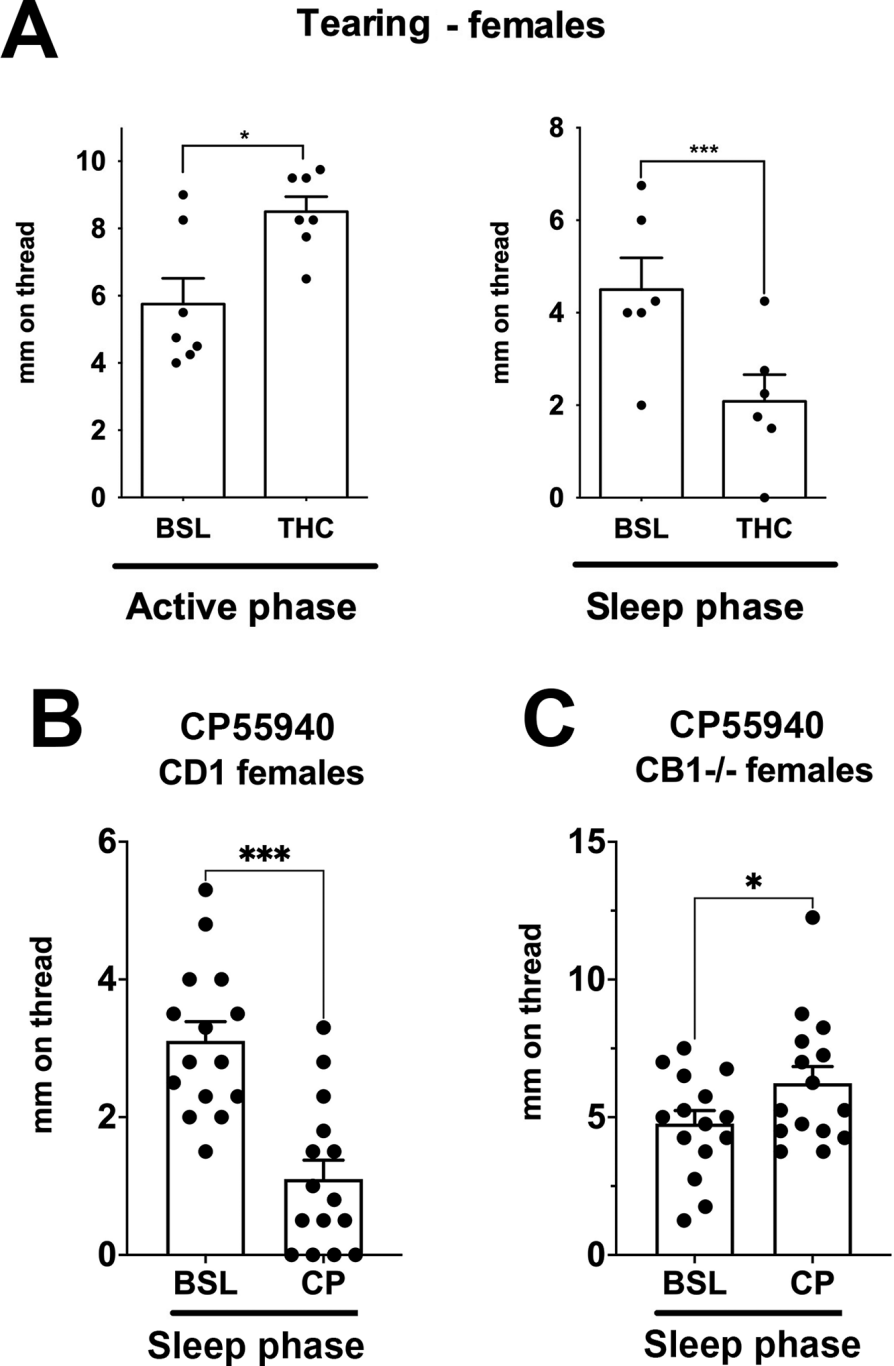
CB1 activation has differential circadian effects on tearing. (**A**) THC increases tearing in females during their active phase, but during their rest phase, THC reduces tearing (*n* = 7, 6). (**B**) CB receptor agonist CP55940 similarly reduces tearing during the rest phase in females (*n* = 15). (**C**) CP55940 does not reduce tearing during the rest phase in CB1 knockout females, instead slightly increasing tearing (*n* = 15). *P < 0.05, ***P < 0.005 by paired *t*-test versus baseline.

We have previously reported that THC and the cannabinoid receptor agonist CP55940 each increase tearing in female mice during their active phase in what appeared to be a CB1-dependent manner since the effect was not seen in CB1 knockout mice.^17^ However, these experiments were done in C57BL/6 mice. Here we tested the effects of THC and CP55940 in CD1 strain female mice. We found that THC (4 mg/kg, IP) similarly increased tearing during the active phase, but additionally found that THC now reduced tearing in the sleep phase (Fig. 2A, *P* = 0.035 vs. control during active phase, *n* = 7; *P* = 0.004 vs. baseline during sleep phase by paired *t*-test, *n* = 6). CP55940 (0.5 mg/kg, IP) reduced tearing by an even greater extent (Fig. 2B, *P* = 0.0002 by paired *t*-test vs. baseline, *n* = 15). This reduction in tearing now resembled the reductions previously reported in males. CP55940 did not decrease tearing in CB1 knockout mice, instead causing a modest but statistically significant increase in tearing (Fig. 2C*,* *P* = 0.034 by paired *t*-test, *n* = 15).

### CB1 Receptors Tonically Inhibit Tearing During the Sleep Phase in Females

**Figure 3.**
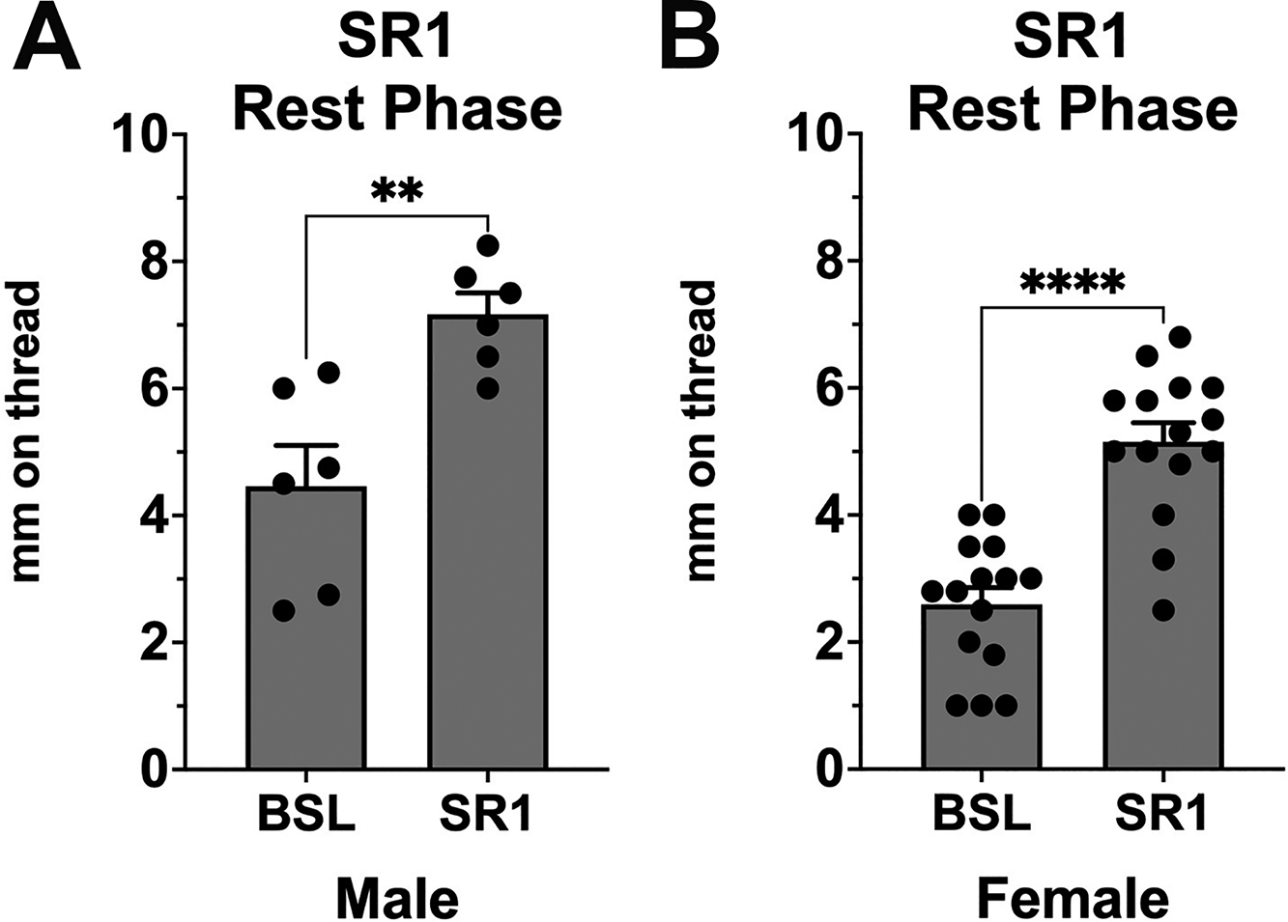
CB1 receptor antagonist increases basal tearing in males and females during the rest phase. (**A**, **B**) The CB1 antagonist SR141716 increases tearing during the sleep phase in both (**A**) males (*n* = 6) and (**B**) females (*n* = 15). **P < 0.01, ****P < 0.0001 by paired test versus baseline.

The reduction of tearing during the sleep phase combined with our finding of exaggerated tearing in CB1 KO females during the sleep phase raised the possibility that CB1 receptors are tonically active in females during the sleep phase. If so, a CB1 receptor antagonist would be predicted to increase tearing during the sleep phase. We had previously found that treatment with the CB1 antagonist in females did not alter tearing during the active phase, although as noted earlier, these were C57BL/6 mice.^17^ We found that SR141716 treatment (4 mg/kg, IP) during the sleep phase increased tearing in both female and male CD1 strain mice (Fig. 3, *P* < 0.01 vs. baseline in males by paired *t*-test, *n* = 6; *P* < 0.001 vs. baseline in females, *n* = 15).

### A Sex-Dependent Difference in CB1 Protein Expression in Female Lacrimal Gland

**Figure 4.**
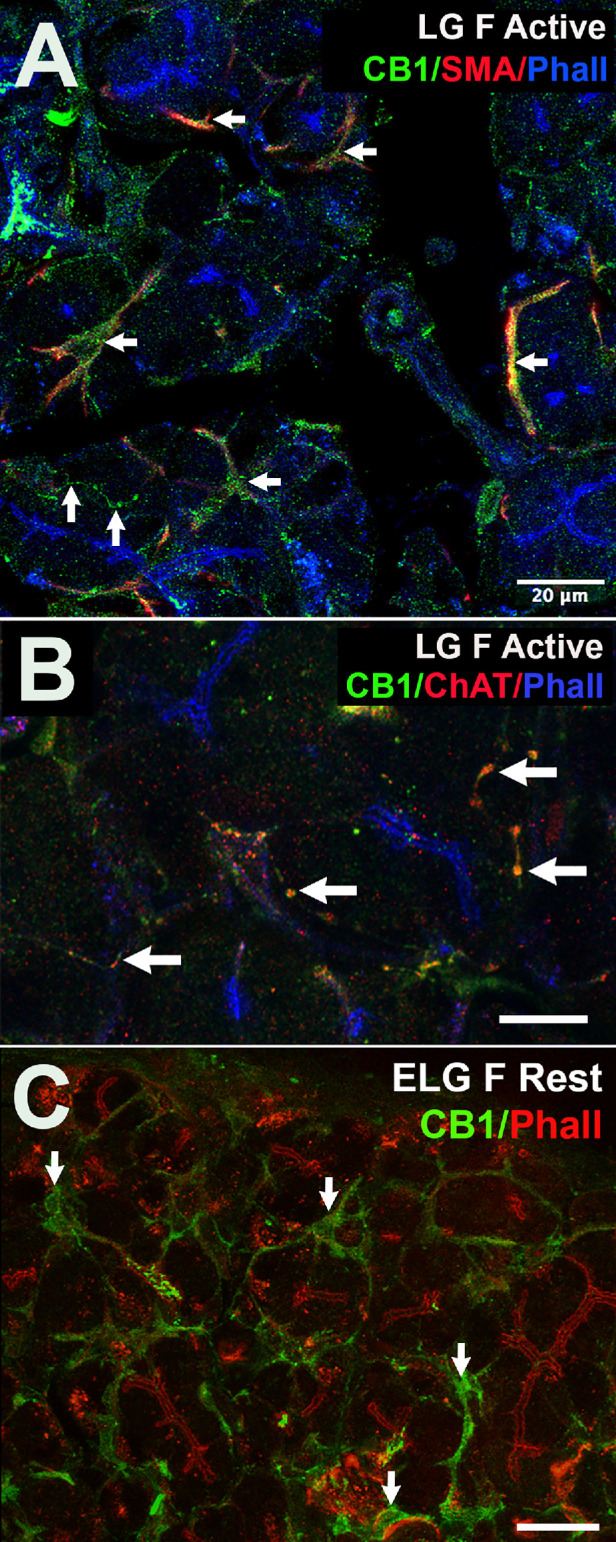
CB1 protein in lacrimal gland of female mice is expressed in myoepithelial cells as well as parasympathetic inputs. (**A**) Lacrimal gland (LG) of female CD1 strain mouse during active phase shows CB1 (*green*) colocalizes with myoepithelial cell marker smooth muscle actin (SMA, *red*); overlap in *yellow* (*left arrows*). F actin marker phalloidin (Phall, *blue*) counterstains intra-acinar ducts. CB1-positive axon-like processes are also seen (*upward arrows*). (**B**) Axon-like CB1 colocalizes with parasympathetic marker ChAT (*red*, *arrows*). (**C**) CB1 staining in tissue from female rest phase lacrimal gland shows similar staining pattern, including myoepithelial cells (*arrows*). Phalloidin counterstain in *red*. *Scale bars*: (**A**) 20 µm, (**B**) 10 µm, and (**C**) 25 µm.

We previously reported expression of CB1 protein in parasympathetic inputs to lacrimal gland of male mice.^17^ Is it possible that the sex differences and/or the circadian variation are reflected in differential expression of CB1 receptors? We tested for CB1 expression in female active-phase mouse lacrimal gland using a previously characterized antibody against CB1 and found that while CB1 is still seen in parasympathetic ChAT-positive axonal inputs (Figs. 4A, 4B), we also see CB1 in myoepithelial cells, colocalized with smooth muscle actin (SMA; Fig. 4A). We also see colocalization of CB1 with myoepithelial cells in tissue from rest phase female mice (Fig. 4C).

## Discussion

We have investigated the sex-dependent interplay of circadian and cannabinoid regulation of tearing in CD1 strain mice. Our chief findings are that both male and female mice exhibit a clear circadian variation in basal tearing, roughly corresponding to higher tearing during the rest phase, albeit with a sex-dependent phase offset wherein male mice peak 5 hours before female mice. The amplitude of WT male cycling is also substantially higher than that of female mice. The cannabinoid signaling system does not appear to affect circadian regulation of tearing in males. However, in females, CB1 receptor deletion or inhibition exaggerates circadian variation in tearing with no apparent change in phase. Functional studies with pharmacological agents and knockouts suggest that endocannabinoids tonically inhibit tearing via CB1 during the rest phase, dampening the oscillations in females. Indeed, the CB1 receptor role may account for the lower amplitude of oscillations seen in females versus males. Protein expression studies in females indicate that CB1 is expressed in parasympathetic inputs, as in males,^17^ but also in myoepithelial cells, a sex-dependent difference that may contribute to the functional differences reported here.

Our findings of a robust circadian variation in basal tearing in mice differ somewhat from those of Vu et al.,^12^ who tested C57BL/6 male mice and saw a ∼75% increase in peak tearing over trough. We avoided the use of that strain in this study since those mice have nonstandard circadian phenotypes due to a melatonin deficiency.^20^ Indeed, in control experiments testing noon versus midnight in C57BL/6 males and females, we observed only modest differences in either sex. Vu et al.^12^ reported peak tearing toward the end of the dark/active phase, this differing somewhat from our finding of peak values at about halfway through the sleep phase. The differences in amplitude and phase may be due to the strain background, but the peak time in the late active phase has been reported in other species such as horse as well as humans.^9^^,^^11^

On the subject of sex differences, the study of horses by Piccione et al.^11^ also compared mares versus stallions and found similar profiles by sex, a contrast with our own findings. Differences in tear production and cannabinoid receptor distribution between species may limit the applicability of these results to human physiology or clinical practice. It will be important to learn whether the situation with mice is species or strain specific or perhaps related to their nocturnal nature.^21^

In the central nervous system, cannabinoid receptors generally play an inhibitory role, often by inhibiting presynaptic neurotransmitter release, reviewed in Kano et al.^22^ We have shown that CB1 is present on presynaptic terminals of cholinergic axons innervating the lacrimal^17^ and submandibular salivary glands^18^ and hypothesize that CB1 activation inhibits the release of the parasympathetic neurotransmitter acetylcholine, but that study did not test for expression in the female lacrimal gland. Our examination of protein expression in the female lacrimal gland was consistent with this but also yielded surprises. We do see CB1 colocalized with parasympathetic terminals stained for ChAT (Fig. 4B), but CB1 is also prominently expressed in myoepithelial cells of the female lacrimal gland, a pattern of expression that is seen regardless of the phase (active versus dark). Myoepithelial cells wrap around acinar clusters in a spiderlike embrace and contribute to the release of fluid from acini in a cAMP-dependent manner.^23^ Activation of G_i/o_-coupled CB1 receptors in myoepithelial cells would be expected to inhibit production of cAMP^24^ and so might reduce the contractive behavior of these cells, but this will require additional investigation.

Our results suggest that female mice experience a tonic activation of CB1 receptors that inhibits tearing during their sleep phase. If so, one would expect an increase in endocannabinoid levels during that phase. The cannabinoid signaling system is unusual in that two candidate ligands have been identified. On the one hand, there is 2-arachidonoyl glycerol^25^ that mediates many effects in neurons,^26^ but arachidonoyl ethanolamine and related acyl ethanolamines are also implicated in cannabinoid function.^27^ Do levels of either of these increase during the rest phase? Our study of salivation pointed to anandamide and the metabolizing enzyme fatty amide hydrolase (FAAH^18^), and a previous study implicated FAAH in the circadian regulation of ocular pressure.^28^ Dissecting the details of endocannabinoid regulation of tearing will be an important future line of inquiry.

Our finding that the CB1 antagonist SR141716 increased tearing in females during the rest phase is consistent with the hypothesized female-specific role. The increase in tearing with a CB1 antagonist under the same conditions in males is somewhat surprising since CB1 knockout males do not see a difference relative to their wild-type strain counterparts. It is possible that CB1 knockout males have a developmental adaptation in circadian tearing. We have noted this for another exocrine gland, with a gradual increase in salivation over the course of about 6 weeks in both male and female CB1 knockouts starting at the time of weaning.^18^

Our finding that CP55940, an agonist at both CB1 and CB2 receptors, caused an increase in tearing in CB1 knockout females differs from our previous findings.^17^ That study had detected a THC- but not CP55940-mediated increase in tearing in CB1 knockout males and no effects of either THC or CP55940 in knockout females. One difference between our previous experiments is that here we devoted considerable attention to prior handling of the mice to reduce the stress response, since that might interfere with a circadian phenotype. This may account for the different results. The current results may be an indication that another cannabinoid-related receptor such as CB2 is active in these mice.

We conclude that CD1 strain mice display a robust sex-dependent circadian variation in tearing that is differentially regulated by cannabinoid CB1 receptors. The complex regulation of tearing in this mouse strain highlights the potential of mice for the study of circadian tearing, with the caveat that several widely used strains have compromised circadian function. Given the substantial variation in tearing (twofold in males), the time of day must be factored into experimental design. The pronounced sex dependence of circadian tearing also highlights the importance of studying both males and females.

It remains unclear whether and, if so, to what extent circadian variations and perturbations of these variations translate to humans and/or affect ocular surface health or stability. Future studies should explore the potential circadian impacts on tear film quality, ocular comfort, or the risk of developing dry eye symptoms.
